# Oral tetracyclines for bone and joint infections: what do we know?

**DOI:** 10.5194/jbji-10-143-2025

**Published:** 2025-04-08

**Authors:** Tom Cartau, Jocelyn Michon, Renaud Verdon, Aurelie Baldolli

**Affiliations:** 1 CHU de Caen, Infectious Diseases Department, Avenue de la Côte de Nacre, Caen, 14000 France; 2 Reference Center for Complex Bone and Joint Infection, Avenue de la Côte de Nacre, Caen, 14000 France; 3 Calvados, Normandie University, UNICAEN, CHU de Caen Normandie, Caen, Normandy, 14000 France; 4 INSERM U1311 DynaMicURe, Normandie University, UNICAEN, UNIROUEN, Caen, France

## Abstract

**Background and aim**: Complex bone and joint infections (BJIs), including prosthetic joint infections (PJIs) and infections associated with osteosynthetic materials, present significant treatment challenges that often require surgical intervention and prolonged antibiotic therapy. In France, the incidence of PJIs in knee and hip arthroplasties ranges from 0.79 % to 2.4 %, with staphylococci being the primary pathogens involved. Recent studies have suggested that oral antibiotic therapy may be as effective as intravenous therapy and that 12 weeks of antibiotic treatment are needed. Tetracyclines, particularly doxycycline and minocycline, are of interest because of their broad-spectrum activities, good oral bioavailability, and potential efficacy in treating BJIs. We aimed to provide a literature review on the role of oral tetracyclines in the management of BJIs.

**Method**: We performed a systematic review of the literature identified via an electronic search of PubMed and ScienceDirect.

**Results**: A total of 648 articles were screened, and 31 studies were included. Pharmacological studies demonstrated that the bone to blood penetration ratio ranged from 0.06 to 0.75. Less than 20 % of strains implicated in BJIs exhibited resistance to oral tetracyclines. Four studies demonstrated potential inhibition of strain growth. Eight studies that included 62 patients reported curative treatment, with a success rate ranging from 82 % to 100 % for PJIs regardless of the surgical management. For suppressive therapy, 10 studies that included 201 patients reported success rates ranging from 57 % to 100 %. The rate of adverse effects ranged from 0 % to 14 % for curative treatment and from 0 % to 57 % for suppressive treatment, leading to treatment discontinuation in less than 20 % of cases.

**Conclusion**: This review highlights that the number of studies supporting the use of oral tetracyclines for the treatment of BJIs is limited. More robust pharmacological and clinical studies are needed to confirm the safety and efficacy profiles of oral tetracyclines for the treatment of BJIs.

## 1 Introduction

Complex bone and joint infections (BJIs), such as prosthetic joint infections (PJIs) or infections associated with fracture-related infections (FRIs), are typically challenging to treat and require surgery, along with a prolonged course of antibiotics (Bernard et al., 2021; Fillingham et al., 2019; McNally et al., 2021; Parvizi et al., 2018). In France, PJIs were reported in 0.79 % to 0.89 % of knee arthroplasties and 1.16 % to 2.4 % of hip arthroplasties in 2021 (Astagneau, 2023). FRIs are more common than PJIs, particularly in cases of open trauma, with the prevalence ranging from 1 % to 30 % (Metsemakers et al., 2018). Staphylococci are responsible for more than half of all PJI or FRI cases (Lemaignen et al., 2021). Managing these BJIs is complex, and guidelines, especially those concerning antimicrobial therapy and administration of antibiotics, vary among countries (Ariza et al., 2017; Ertel-Pau, 2014; Osmon et al., 2013). A study on more than 1054 participants suggested that oral therapy is as effective as intravenous therapy during the first 6 weeks (Li et al., 2019). Additionally, recent research revealed a higher failure rate in PJI patients on a 6-week antibiotic course than in those on a 12-week course (Bernard et al., 2021). The usual oral antibiotic course for BJIs involves a combination of antibiotics, especially for staphylococcal infections. A recommended treatment for staphylococcal BJIs is quinolone and rifampicin in combination with first-line antibiotherapy, when possible (Ertel-Pau, 2014; Osmon et al., 2013). Treatments containing rifampicin have demonstrated a greater efficacy than those without rifampicin (El Helou et al., 2010). However, these treatments can lead to adverse events (AEs), such as musculoskeletal issues, drug interactions, and nausea, resulting in drug discontinuation or the need to change antibiotics in up to 20 % of cases (El Helou et al., 2010; Nguyen et al., 2015; Shah et al., 2020). In the cases of side effects or nonsusceptible bacteria, cotrimoxazole, linezolid, tedizolid, macrolides, lincosamides, streptogramins, or cyclins can be used as oral alternatives (Osmon et al., 2013).

Tetracyclines are a group of bacteriostatic antibiotics that inhibit bacterial protein synthesis through interactions with the 30S subunit of bacterial ribosomes. Tetracyclines have broad spectra of activity against both gram-positive and gram-negative bacteria and are often used to treat atypical infections, such as brucellosis, rickettsiosis, Q fever, Lyme disease, sexually transmitted infections, and malaria (Bahrami et al., 2012). Tetracyclines can be categorized into three groups on the basis of their pharmacokinetic and antibacterial properties. Group 1 includes older agents, such as tetracycline and rolitetracycline, which have a reduced oral bioavailability (77 %–88 %). Group 2 comprises doxycycline and minocycline, which are more lipophilic than older tetracyclines and have an oral bioavailability of approximately 95 %–100 %; this improves their tissue distribution. Both drugs have a prolonged half-life (12–16 h) and have shown sufficient antistaphylococcal activity, including that against methicillin-resistant *Staphylococcus aureus* (MRSA) (Ruhe et al., 2005). Protein binding varies from 76 % to 93 %. Group 3 involves tigecycline, which is administered intravenously and is more commonly used for multidrug-resistant bacterial infections, and omadacycline and eravacycline (Agwuh and MacGowan, 2006; Bahrami et al., 2012; Bidell and Lodise, 2021). Tetracycline antibiotics chelate calcium and are found in the cytoplasm of osteoclasts, which explains their affinity for bones (Donahue et al., 1992; Warner et al., 2022). These antibiotics have few side effects (namely, photosensitivity and gastrointestinal effects) and are well-tolerated. Owing to their broad spectra of activity, good oral bioavailability, and prolonged activity, doxycycline and minocycline are potential options for the oral treatment of BJIs. However, data concerning the efficacy of oral cyclins against BJIs due to common bacteria are limited. The aim of this work was to provide a literature review on the role of oral tetracyclines in the management of BJIs.

## 2 Materials and methods

We performed a systematic review of the literature identified via an electronic search of PubMed and ScienceDirect by using the following keywords: “tetracycline” OR “doxycycline” OR “minocycline” AND “bone and joint infection” OR “periprosthetic joint infection” OR “osteomyelitis” OR “synovial fluid” OR “bone penetration” OR “joint penetration” (MeSH). No language or age constraints were applied to the search. Databases was searched between 1960 and November 2024. Articles were selected for review if their titles or abstracts suggested the use of oral tetracyclines against bone and joint infections caused by common microorganisms or if the titles suggested data on bone penetration of tetracyclines. Veterinary articles or articles concerning intracellular microorganisms, zoonotic diseases (such as Whipple's disease, Lyme disease, and Q fever), sexually transmitted infections, or intravenous tetracyclines were excluded. Case reports, case series, and cohort studies were included only if data regarding clinical presentation, microorganisms, treatment, and outcomes were available. Special attention was given to avoiding the inclusion of duplicate cases among meeting abstracts, case reports, or articles. Suppressive antibiotic treatment (SAT) refers to the long-term administration of antibiotics with the aim of reducing symptoms and delaying the progression of BJIs. Curative antibiotic treatment refers to an antibiotic regimen with the aim of curing BJIs, often in combination with surgical management. This literature review was conducted according to the PRISMA (Preferred Reporting Items for Systematic Reviews and Meta-Analyses) guidelines (Page et al., 2021).

## 3 Results

**Figure 1:**
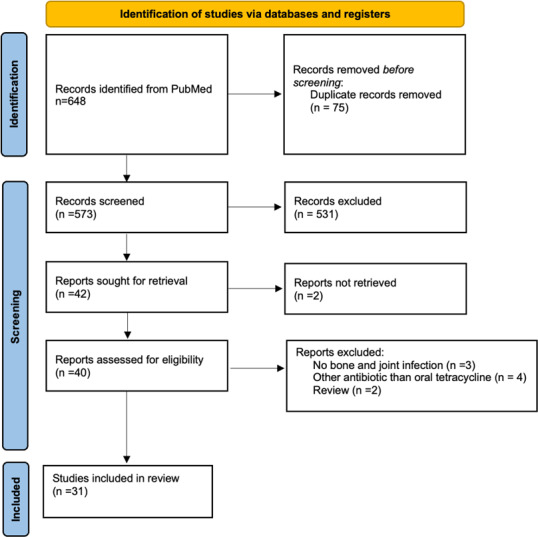
Flowchart of the selection process of the 31 studies included in this review on oral tetracycline use for bone and joint infections.

The search of the PubMed and ScienceDirect databases yielded a total of 648 citations. After excluding duplicates and articles whose titles and abstracts did not meet the inclusion criteria, 40 full-text articles were assessed for eligibility. Finally, 31 studies, mostly case series and retrospective studies, were included (Fig. 1): 3 studies on bone penetration, 5 studies on susceptibility profiles, 4 on the effect of oral tetracyclines on biofilm formation, 1 pharmacological study on an animal model, 8 on curative treatment, and 10 on suspensive treatment.

### 3.1 Tetracycline, doxycycline, and minocycline penetration into bone and joints

Three studies focused on oral tetracyclines into bone and joints.

In the study by Bystedt et al. (1976), 30 patients received either doxycycline (200 mg, n=10 ), tetracycline (500 mg, n=10 ), or oxytetracycline (250 mg, n=10 ) orally for mandibular osteitis just before surgery (dental extraction). The serum peak concentration of doxycycline was greater than that of tetracycline or oxytetracycline (4.4 [1.9–6.8] µg mL^−1^ versus 2.3 [1.1–4.3] µg mL^−1^ and <2 µg mL^−1^, respectively). The dental alveolar concentration of doxycycline was approximately 1.0 µg mL^−1^ lower than its serum concentration (75 % of bone penetration) and higher than the dental alveolar concentrations of oxytetracycline and tetracycline (0.2 µg mL^−1^ and 0.5 µg mL^−1^ lower than the serum concentrations, respectively) (Bystedt et al., 1976). In another study, 34 patients were treated with 200 mg of intravenous doxycycline 2 h before orthopedic surgery for fracture. At 3 h post-administration (peak doxycycline concentration in the serum), the mean serum concentration in 14 females was 8.3 [5.4–11.5] µg mL^−1^, whereas the mean bone concentration was 0.13 [0–0.51] µg mL^−1^. For 13 males, the mean serum concentration was 5.97 [4.7–8.8] µg mL^−1^, and the mean bone concentration was 0.11 [0–0.36] µg mL^−1^ (Gnarpe et al., 1976). In more recent studies, after administration of 200 mg of intravenous doxycycline to 25 patients undergoing orthopedic surgery, the bone concentrations ranged from 0.1 to 2.2 µg g^−1^ depending on the method used (electrophoresis or agar diffusion) (Dornbusch, 1976).

### 3.2 Susceptibility profiles of microorganisms involved in BJIs to tetracyclines

Five studies focused on susceptibility profiles of microorganisms involved in BJIs to tetracyclines.

Less than 20 % of staphylococcal strains are resistant to tetracycline, but the resistance is greater for MRSA and coagulase-negative *Staphylococcus* (CoNS) strains and for enterococcal ( > 40 %) strains (Citron et al., 2014; Duployez et al., 2022; Hamad et al., 2015; Pfaller et al., 2018). BJI-causing strains that were collected in the USA, Europe, Türkiye, Ukraine, Russia, and Israel presented the following rates of resistance to tetracycline: 5.4 % and 6.1 % among 801 *S. aureus* strains, 3.2 % and 3.7 % among 534 MSSA strains, 9.8 % and 10.9 % among 267 MRSA strains, 13.1 % and 15 % among CoNS strains, 51.5 % and 55.2 % among 164 β -hemolytic streptococcal strains (according to the CLSI and EUCAST break points, respectively), and 75.6 % (according to the CLSI break point only) among *Enterococcus faecalis* strains (Pfaller et al., 2018). In another large retrospective study that was conducted at a French reference center for complex BJIs to describe the antibiotic susceptibility profiles of bacteria isolated from BJIs over 10 years, resistance to tetracycline for staphylococci remained quite stable (26.6 %, 917 out of 3449 strains). Among all CoNS strains ( n=2373 ), 35.4 % were resistant to tetracycline and 2.1 % were resistant to minocycline, whereas among *S. aureus * strains ( n=1101 ), only 11 % were resistant to tetracycline and 3.7 % were resistant to minocycline (Duployez et al., 2022) A total of 26 gram-positive isolates from PJI patients who were not susceptible to doxycycline were evaluated for minocycline susceptibility using gradient diffusion test strips. *E. faecium* strains ( n=5 ) were all resistant to minocycline, whereas 40 % (2 out of 5) of *E. faecalis* strains, 72.7 % (8 out of 11) of MRSA strains, and 100 % (5 out of 5) of CoNS were susceptible to minocycline while being resistant to doxycycline (Doub et al., 2022).

## 4 Tetracyclines and biofilm formation activity in BJIs

Four studies focused on the activity of oral tetracycline against bacterial biofilm formation in BJIs.

Budge et al. (2020) compared the efficacies of vancomycin, doxycycline, and penicillin against the planktonic and 72 h old biofilm forms of *Cutibacterium acnes* strains causing PJIs. The data revealed that doxycycline was effective in eradicating both planktonic and 72 h old biofilm forms of *C. acnes*, as indicated by its minimum inhibitory concentration (MIC) and minimum bactericidal concentration (MBC) values. The effective antibiotic concentrations ranged from 1 to 1000 µg mL^−1^, but only doxycycline achieved inhibitory and bactericidal concentrations across all the tested strains. However, notably, the MIC and MBC values for biofilms were greater than those for the planktonic form ( p<0.05 ) for all antibiotics, including doxycycline (Budge et al., 2020).

In another study, Mandel et al. (2019) analyzed the activities of 10 important antibiotics (cefazolin, clindamycin, vancomycin, rifampicin, linezolid, nafcillin, gentamicin, trimethoprim/sulfamethoxazole, doxycycline, and daptomycin) against PJI-associated *S. aureus* strains that were grown as 48 h old biofilms and planktonic cultures. Both MSSA and MRSA biofilms demonstrated decreased sensitivity to all clinically used antibiotics, but only rifampicin, doxycycline, and daptomycin had significant activity against 48 h old biofilms, with MBCs ranging from 80 to 2000 µg mL^−1^. A total of 90 % of *S. aureus* biofilms could be killed by rifampicin, 50 % by doxycycline, and only 15 % by daptomycin (Mandell et al., 2019).

In a study on the antibiofilm activity of doxycycline, Koch et al. (2024) compared the activities of antibiotics against 48 h old biofilms with those against planktonic cultures of PJI-associated *S. epidermidis* strains. Only rifampicin and doxycycline had a significant effect on biofilm formation and were able to eradicate 64 % and 18 % of *S. epidermidis* biofilms, respectively, with MBCs ranging from 32 to 2000 µg mL^−1^. However, the MBCs were greater for doxycycline than for rifampicin, and biofilm-forming strains were more resistant to doxycycline (Koch et al., 2020).

In a more recent study, the combination of doxycycline and rifampicin was able to eradicate biofilms of one-third of *S. aureus* strains, whereas the combination of doxycycline and moxifloxacin was ineffective. These results favor a synergistic in vitro effect of doxycycline and rifampicin (Perez-Alba et al., 2023).

In these four studies, all the biofilm-producing strains of *C. acnes*, *S. epidermidis*, and *S. aureus* demonstrated greater tolerance to antibiotics than planktonic cultures.

## 5 Use of oral tetracyclines against BJIs: an animal study

Only one study has evaluated the effect of oral doxycycline in an animal model of BJI.

The efficacies of oral and intravenous monotherapies and combination antibiotic–rifampicin regimens were evaluated in a mouse model of MRSA-induced hip PJI. A total of 2 weeks after bacterial inoculation (to allow the formation of biofilm), different antibiotic therapies, including oral doxycycline, were administered for 6 weeks ( n=10 per group). The bacterial burden was assessed by in vivo bioluminescent imaging and ex vivo counting of colony-forming units (CFUs). The effect of doxycycline monotherapy on bioluminescence imaging signals was modest compared with that of the antibiotic–rifampicin regimen. All monotherapy treatments (including oral doxycycline) failed to clear the infection, whereas oral linezolid–rifampicin and all intravenous antibiotic–rifampicin combinations resulted in no viable bacteria (no CFUs). The percentage of tissue with CFUs present out of the total number of samples assayed was the same in the doxycycline group as in the control without antibiotics. The combination of doxycycline and rifampicin was not tested (Thompson et al., 2017).

## 6 Clinical data on the use of oral tetracyclines against BJIs

### 6.1 Oral tetracyclines as curative treatments for BJIs

No randomized studies have evaluated the efficacies of oral tetracyclines in the curative treatment of BJIs. Only eight studies were found in the literature, including two prospective studies (Bart et al., 2020; Clumeck et al., 1984; Jang et al., 2024; Matt et al., 2021; Norden et al., 1983; Preininger, 1973; Ruhe et al., 2005; Sato et al., 2019). A total of 62 cases, including 13 cases of osteomyelitis, 1 case of arthritis, and 48 cases of prosthetic joint infection (PJI), were described. The oral tetracyclines used were minocycline in 45 patients (73 %), doxycycline in 3 patients (5 %), and an unspecified tetracycline in 14 patients (23 %). Oral tetracyclines were combined with rifampicin in 12 patients (19 %), trimethoprim/sulfamethoxazole in two patients (3 %) (Ruhe et al., 2005; Sato et al., 2019), dalbavancin in 3 patients (5 %), and vancomycin in 34 patients (55 %). In the study by Bart et al. (2020), the efficacy of a vancomycin and minocycline regimen was compared with that of a vancomycin and rifampicin regimen. The relapse and reinfection rates did not differ between the two groups. New infections seemed to be more common in the minocycline group, but the difference was not significant (6 vs. 3; p=0.3 ) (Bart et al., 2020).

Infections were caused by *S. aureus* in 20 patients (32 %) and by coagulase-negative staphylococci in 39 patients (63 %). No microbiological data were available for the remaining three patients (5 %). There were 10 (16 %) treatment failures and 4 (6 %) treatment discontinuations due to adverse events (AEs). In the cases of treatment failure, the infection was due to the same pathogen in five patients (8 %) and to another pathogen in two patients (3 %). No data were available for the remaining three patients (5 %). For osteomyelitis, the duration of treatment ranged from 5 d to 14 months, with a success rate of 62 % (8 out of 13 patients). For PJI, the treatment duration ranged from 11.4 weeks to 90 d, with a success rate of 91 % (41 out of 45 patients), when data were available (Bart et al., 2020; Clumeck et al., 1984; Jang et al., 2024; Norden et al., 1983; Preininger, 1973; Ruhe et al., 2005; Sato et al., 2019).

The results of these studies are detailed in Table 1.

**Table 1:** Summary of eight studies on the use of oral tetracyclines for curative antibiotic treatment of bone and joint infections.

	Treatment	BJI	Microorganisms	Mean of follow-up/mean duration of treatment	Side effects	Outcomes
Preininger (1973) Case report, n=1	Minocycline	Osteomyelitis, n=1	MSSA, n=1	NA	NA	Success, n=1 (100 %) **Failure**, n=0(0%)
Norden et al. (1983) Prospective study, n=14	Rifampicin 600 mg qd + doxycycline 100 mg bid, n=3	Osteomyelitis, n=3	SA, n=3	NA/6 months	NA	Success, n=1 (33 %) after 5 years of follow-up Failure, n=2 (67 %) due to other bacteria
Clumeck et al. (1984) Retrospective study, n=25	Minocycline 100 or 200 mg bid + rifampicin 300 mg bid n=4	Osteomyelitis, n=4	SA, n=4	NA/22 (5–119) d	None	Failure, n=1 after 3 months of treatment due to the appearance of rifampicin and minocycline resistance
Ruhe et al. (2005) Retrospective study, n=24	Minocycline 100 mg bid + rifampicin, n=3 or minocycline 100 mg bid + trimethoprim/ sulfamethoxazole, n=1	Osteomyelitis, n=4 Arthritis, n=1	MRSA, n=5	NA	2/24 (8 %) nausea or vomiting, leading to treatment discontinuation	Success, n = 21 (87.5 %) Failure, n=3 (12.5 %)
Sato et al. (2019) Retrospective study, n=1	Minocycline 100 mg qd + trimethoprim/ sulfamethoxazole 6 g qd	Wrist osteomyelitis, n=1	MRSA, n=1	1 year/14 months	0	Success, n=1 (100 %) **Failure**, n = 0 (0 %)
Bart et al. (2020) Prospective study, n=34	Minocycline 100 or 200 mg bid + vancomycin 6–8 weeks, n=34	Hip PJI, n=22 Knee PJI, n=12	MRCoNS, n=34	43 months/85 (median) (84–90) d	n=3/22 (14 %) leading to two treatment discontinuations (hepatitis and thrombocytopenia)	Success, n = 32 (94 %) Failure, n=2 (6 %)
Matt et al. (2021) Retrospective study, n=16	Tetracycline (nonspecified), n=3	PJI, n=3	NA	NA	0	No data
Jang et al. (2024) Retrospective study, n=24	Doxycycline or minocycline (no details), n=11	Hip PJI, n=3 Knee PJI, n=6 Shoulder PJI, n=2	MRSA, n=4 MSSA, n=2 CoNS, n=5	1 year/11.4 weeks	NA	Success, n=9 (82 %) Failure, n=2 (18 %)

SA: *Staphylococcus aureus; * MSSA: methicillin-susceptible *Staphylococcus aureus; * MRSA: methicillin-resistant *Staphylococcus aureus*; MRCoNS: methicillin-resistant coagulase-negative *Staphylococcus;* PJI: prosthetic joint infection; qd: once a day; bid: twice a day; NA: not available

### 6.2 Oral tetracyclines as a suppressive antibiotic therapy (SAT) for BJIs

Oral tetracyclines are more likely to be used as an SAT against BJIs. A total of 10 retrospective studies have reported the efficacies of SATs, including oral tetracyclines, in patients with PJIs (Ceccarelli et al., 2023; Jang et al., 2024; Leijtens et al., 2019; Pradier et al., 2018; Prendki et al., 2014; Rao et al., 2003; Sandiford et al., 2020; Segreti et al., 1998; Siqueira et al., 2015; Wouthuyzen-Bakker et al., 2017). No prospective study was found. These studies reported 201 BJIs, including 70 hip PJIs (35 %), 65 knee PJIs (32 %), two shoulder PJIs (1 %), four elbow PJIs (2 %), and five fracture-related bone infections (2 %). No data on anatomical sites were available for 55 patients (27 %).

Staphylococci were the main bacteria involved in these BJIs ( n=193 , 96 %). Other implicated bacteria are detailed in Table 2.

**Table 2:** Summary of 10 studies on the use of oral tetracyclines for suppressive antibiotic treatment of bone and joint infections.

	Treatment	BJI	Microorganisms	Mean of follow-up/mean duration of SAT (years)	Side effects	Outcomes at the end of follow-up
Segreti et al. (1998) Retrospective study, n=18	Total: n= 5 Minocycline 100 or 200 mg qd + rifampicin 600 mg qd	Hip PJI, n= 1 Knee PJI, n= 4	CoNS, n= 3 MRSA, n= 2	5/4.1	2/5 (40 %) Diarrhea, n= 2	Success, n= 5 (100 %) Failure, n = 0 (0 %)
Rao et al. (2003) Retrospective study, n= 36	Total: n= 14 Minocycline 100 mg d^−1^, n= 2 Minocycline 100 mg + rifampicin 600 mg, n= 11 Doxycycline 200 mg qd + amoxicillin 500 mg tid, n= 1	No data	MRCoNS/MRSA, n= 14 *Enterococcus*, n= 1	5.1/4.3	No data	Success, n= 12 (85 %) Failure, n= 2 (15 %)
Prendki et al. (2014) Retrospective study, n= 32	Doxycycline 200 mg qd, n= 1	No data	MRSA, n= 1	No data	No data	Success, n= 1 (100 %)
Siqueira et al. (2015) Retrospective study, n= 92	Total: n= 40 Minocycline 100 mg qd, n= 1 Doxycycline 100 mg qid, bid, or qd, n= 39	No data	MRSA, n= 8 MSSA, n= 7 MSCoNS, n= 8 MRCoNS, n= 12 *Enterococcus*, n= 1 *Streptococcus*, n= 1 P . *acnes*, n= 1 Diphtheroid-like bacilli, n= 1 Negative culture, n= 1	5.8/5.3	No data	Success, n= 63 (68.5 %) No specific data on the oral tetracycline group
Wouthuyzen-Bakker et al. (2017) Retrospective study, n= 21	Total: n= 7 Doxycycline 100 mg qd, n= 1 Minocycline 200 mg qd, n= 4 100 mg qd, n= 2	Hip PJI, n= 6 Knee PJI, n= 1	SE, n= 6 SA, n= 1 *B. fragilis*, n= 1 *E. cloacae*, n= 1	1.8 (median)/1.8	4/7 (57 %) – Phototoxicity, n= 2 – Nausea, n= 2 – Diarrhea, n= 1	Success, n= 6 (85 %) Failed, surgical intervention needed: n= 1
Pradier et al. (2018) Retrospective study, n= 78	Total: n= 78 Minocycline 200 mg qd, n= 6 Doxycycline 200 mg qd, n= 72	Hip PJI, n= 35 Knee PJI, n= 37 Elbow PJI, n= 4 Shoulder PJI, n= 2	*S. aureus*, n= 42 CoNS, n= 33 *Streptococcus*, n= 12 *Enterococcus*, n= 3 Enterobacteria, n= 5 *P. aeruginosa*, n= 1 Anaerobes, n= 7 Others, n= 4	2.8/1.8	14/78 (18 %) leading to SAT discontinuation in six patients (8 %) due to phototoxicity – Phototoxicity, n= 7 – Nausea, n= 7 – Pruritus, n= 2 - Vertigo, n= 2	Success, n= 56 (72 %) Failure, n= 22 (28 %)
Leijtens et al. (2019) Retrospective study, n= 23	Total: n= 14 Doxycycline 100 mg qd, n= 11 200 mg qd, n= 3	Hip PJI, n= 14	CoNS, n= 11 SA, n= 4 *P. acnes*, n= 2 *C. perfringens*, n= 1 *P. aeruginosa*, n= 2 *Corynebacterium*, n= 1 GPR, n= 1	2.7/3.1	3/14 (21 %) leading to SAT discontinuation in one patient – Pruritus, n= 1 – Nausea, n= 1 – Thrombocytopenia, n= 1	Success, n= 8 (57 %) Failure, n= 6 (43 %)
Sandiford et al. (2020) Retrospective study, n= 26	Total: n= 13 Doxycycline, n= 13	Hip PJI, n= 7 Knee PJI, n= 6	SE or CoNS, n= 6 SA, n= 6 *P. acnes*, n= 1 *S. dysgalactiae*, n= 1	3.2/3.1	No side effects	Success, n= 12 (92 %) Failure, n= 1 (8 %) with surgical intervention needed
Ceccarelli et al. (2023) Retrospective study, n= 16	Minocycline 200 mg qd, n= 16	Hip PJI, n= 6 Knee PJI, n= 5 Fracture-related infection, n= 5	MRSA, n= 2 CoNS, n= 14 (including one co-infection with *E. coli*)	1.75 (median)/1.75	3/16 (18.7 %) leading to SAT discontinuation – Teeth staining n= 1 – Epigastric pain, n= 2	Success, n= 10 (62.5 %) Failure, n= 6 (37.5 %)
Jang et al. (2024) Retrospective study, n= 24	Doxycycline or minocycline, no details, n= 13	Knee, n= 12 Hip, n= 1	MRSA, n= 2 MSSA, n= 3 CoNS, n= 8	1/no data (at least 1 year)	No data	Success, n= 13 (100 %) Failure, n= 0 (0 %)

SA: *Staphylococcus aureus; * MSSA: methicillin-susceptible *Staphylococcus aureus; * MRSA: methicillin-resistant *Staphylococcus aureus*; MRCoNS: methicillin-resistant coagulase-negative *Staphylococcus*; MSCoNS: methicillin-susceptible coagulase-negative *Staphylococcus*; SE: *Staphylococcus epidermidis;* CoNS : coagulase-negative* Staphylococcus;* *P. acnes*: *Propionibacterium acnes;* *E. coli: Escherichia coli;* *P. aeruginosa: Pseudomonas aeruginosa;* *C. perfringens:* *Clostridium perfringens;* *E. cloacae: Enterobacter cloacae;* *B. fragilis: Bacteroides fragilis;* *S. lugdunensis: Staphylococcus lugdunensis; S. warneri: Staphylococcus warneri; E. faecalis: Enterococcus faecalis;* *C. koseri: Citrobacter koseri;* GPR: gram-positive rod; PJI: prosthetic joint infection; qd: once a day; bid: twice a day; tid: three times per day; qid: four times per day.

Minocycline was used in 47 patients (23 %), whereas doxycycline was used in 141 patients (70 %). For 13 patients (6 %), the oral tetracycline used was not specified. Minocycline was used in combination with rifampicin in 16 patients (8 %). The median duration of intensive treatment ranged from 1 to 5.3 years, when data were available. The AE rates ranged from 18 % to 57 %, with only 10 treatment discontinuations (5 %) due to AEs reported. The success rates of SAT during the follow-up ranged from 57 % to 100 %. Pradier et al. (2018) documented three strains with acquisition of doxycycline resistance. No other examples of doxycycline resistance were described (Pradier et al., 2018).

The results of these studies are detailed in Table 2.

In a retrospective study of 39 cases of knee or hip PJIs associated with *Staphylococcus aureus*, SAT with doxycycline achieved an 85 % rate of success in patients with initial surgical management (debridement and antibiotherapy with implant retention – DAIR – or implant exchange), with a mean event-free period of 994 d (Pradier et al., 2017). Failure was mostly associated with doxycycline-susceptible bacteria (8 out of 10, 80 %). This is the largest series reported to date on the use of oral tetracycline as an SAT to treat *Staphylococcus aureus*-induced BJIs, and data are reported in another large study (Pradier et al., 2018).

## 7 Discussion

This review provides a comprehensive examination of the use of oral tetracyclines for the treatment of common BJIs. To our knowledge, this is the first review on the use of oral tetracyclines for the treatment of BJIs. We highlight the lack of robust data, with only a few published studies, 8 of which were on curative treatment and 10 on SAT. Most of these studies are retrospective, with only two prospective studies and no randomized trials.

Pharmacological studies on the penetration of oral tetracyclines into bone and joint tissues are scarce, with only three studies available and none published after 1980. These studies present conflicting results, with doxycycline bone penetration ranging from 1.5 % to 75 % (Bystedt et al., 1976; Dornbusch, 1976; Gnarpe et al., 1976). However, variability in methodology, including differences in antibiotic administration, tetracycline concentrations in different bone tissues (cortical vs. cancellous or not specified, pathologic bone vs. normal bone), and measurement techniques (doxycycline-binding protein fraction or doxycycline-binding and not binding fractions and different techniques), likely contributes to these discrepancies.

The strains that are commonly found in BJIs are generally sensitive to doxycycline and minocycline. However, cases of emerging resistance have been reported. In such instances, testing for sensitivity to minocycline is recommended. The mechanisms of resistance are diverse (e.g., efflux pumps, enzymatic degradation, or target mutations) and do not necessarily confer cross-resistance to other tetracyclines (Grossman, 2016).

Four studies have investigated the potential effects of oral tetracycline on biofilm-forming strains of *C. acnes* or *Staphylococcus* spp. implicated in BJIs. The results showed that the effects of doxycycline and minocycline on *S. aureus* biofilms were similar to those of rifampicin and fluoroquinolones. In addition to BJI biofilms, Cerca et al. (2005) reported that compared with cefazolin, vancomycin and dicloxacillin, tetracycline and rifampicin were the two most effective antibiotics for killing bacteria in biofilms (formed in batch and fed-batch modes) (Cerca et al., 2005). These data are supported by those of Monzon et al. (2020), who demonstrated that compared with vancomycin, clindamycin, cephalothin, teicoplanin, and ofloxacin, rifampicin and tetracycline had greater killing effects on *Staphylococcus epidermidis* in biofilms (Monzón et al., 2002). Takahashi et al. (2006) demonstrated that doxycycline inhibited biofilm formation by *Prevotella intermedia*, whereas tetracycline, minocycline, and ofloxacin did not (Takahashi et al., 2006). Despite these promising in vitro data, clinical trials are needed to confirm the clinical relevance of these findings for BJI biofilms.

Oral tetracyclines are recommended as options for SAT in international guidelines and are considered alternatives to standard curative treatments (Osmon et al., 2013). However, these recommendations are based on few high-quality studies with good methodologies. There are no randomized or comparative studies. In this review, we found a failure rate of 16 % (10 out of 61) in cases of curative treatment and a recurrence or treatment failure rate of 0 % to 43 % in cases of suppressive antibiotic therapy. Most studies on the curative treatment of BJIs involved few patients, and the results should be interpreted with caution. Another significant issue is the lack of precision regarding the treatment duration and surgical management, which limits the interpretation of success or failure. For patients experiencing relapse after first-line treatment, surgical management is often not adequately addressed. In the reviewed studies, doxycycline was mostly used in combination but with no comparator. Several studies were excluded despite the use of doxycycline or minocycline orally because of a lack of the description of cases, success, or surgical management. There is most likely an underreporting of patients with BJIs treated with doxycycline. Indeed, in our reference center, doxycycline is an antibiotic that is regularly used, and owing to the lack of studies with larger sample sizes and comparators, it is difficult to assess its effectiveness as a first-line treatment and its place among the different therapeutic options (in combination or as monotherapy and curative treatment or SAT).

Only a few AEs are associated with oral tetracycline, even during prolonged treatment. We found 37 AEs reported, which led to 17 discontinuations of SAT or CAT. This represents 8 % to 57 % of cases with reported AEs. Treatment discontinuations due to AEs ranged from 7 % to 19 % when data were available. The rate of AEs was higher for SAT than for curative treatment. This may be explained by the duration of treatment. AEs, including phototoxicity and gastrointestinal issues, are often moderate. Severe phototoxicity, hepatotoxicity, and cardiological AEs are described less frequently. The rates of adverse effects are similar to those reported with the prolonged use of doxycycline in the treatment of acne, rosacea, and lymphangioleiomyomatosis or in the prevention of sexually transmitted infections (STIs) (approximately 7 % in four studies) (Molina et al., 2018; Moore et al., 2015; Pimenta et al., 2013; Thiboutot et al., 2009).

Rifampicin is known to be an enzymatic inducer at the hepatic level, and doxycycline undergoes at least partial metabolism in the liver. Few studies have investigated the potential impact of this combination on doxycycline blood levels. This combination leads to a decrease in doxycycline blood levels and a reduction in its half-life. This decrease and its impact on the clinical course of BJIs remain to be explored (Bessard et al., 1983; Colmenero et al., 1994; Garraffo et al., 1988).

Compared with other antibiotic regimens that are commonly used for BJIs, oral tetracyclines have very good safety profiles. In a recent retrospective study, AEs occurred in 51 % of patients treated with levofloxacin, leading to treatment discontinuation in 35.6 % of the patients (Vollmer et al., 2021). AEs were observed in 57 % of patients treated with linezolid for BJIs, resulting in treatment discontinuation in 26 % of the cases (Veerman et al., 2023). Rifampicin, which is often used in combination therapy for BJIs because of its efficacy against biofilms, was associated with a 22 % rate of treatment discontinuation due to AEs in another retrospective study (Tonnelier et al., 2021). Oral tetracycline appears to be a good candidate for prolonged oral antibiotic treatment because of its safety profile.

In conclusion, oral tetracyclines could be attractive alternative antibiotics for the management of BJIs because of their antibiofilm activity, good tolerability, and promising preliminary results from clinical studies. However, the current body of evidence is based on studies with methodological limitations, and there is an urgent need for well-designed prospective studies (bone penetration and treatment of BJIs) to confirm these findings and optimize treatment protocols.

## Data Availability

The data that support the findings of this study are not openly available for reasons of sensitivity and data protection.

## References

[bib1.bib1] Agwuh KN, MacGowan A (2006). Pharmacokinetics and pharmacodynamics of the tetracyclines including glycylcyclines. J Antimicrob Chemoth.

[bib1.bib2] Ariza J, Cobo J, Baraia-Etxaburu J, Benito N, Bori G, Cabo J, Corona P, Esteban J, Horcajada JP, Lora-Tamayo J, Murillo O, Palomino J, Parra J, Pigrau C, Del Pozo JL, Riera M, Rodríguez D, Sánchez-Somolinos M, Soriano A, Del Toro MD, De La Torre B (2017). Executive summary of management of prosthetic joint infections. Clinical practice guidelines by the Spanish Society of Infectious Diseases and Clinical Microbiology (SEIMC). Enferm Infec Micr Cl.

[bib1.bib3] Astagneau P (2023). Rapport national de la surveillance semi-automatisée des infections du site opératoire en chirurgie – Données 2020 et 2021.

[bib1.bib4] Bahrami F, Morris DL, Pourgholami MH (2012). Tetracyclines: drugs with huge therapeutic potential. Mini-Rev Med Chem.

[bib1.bib5] Bart G, Zeller V, Kerroumi Y, Heym B, Meyssonnier V, Desplaces N, Kitzis MD, Ziza JM, Marmor S (2020). Minocycline Combined with Vancomycin for the Treatment of Methicillin-Resistant Coagulase-Negative Staphylococcal Prosthetic Joint Infection Managed with Exchange Arthroplasty. J Bone Joint Infect.

[bib1.bib6] Bernard L, Arvieux C, Brunschweiler B, Touchais S, Ansart S, Bru J-P, Oziol E, Boeri C, Gras G, Druon J, Rosset P, Senneville E, Bentayeb H, Bouhour D, Le Moal G, Michon J, Aumaître H, Forestier E, Laffosse J-M, Begué T, Chirouze C, Dauchy F-A, Devaud E, Martha B, Burgot D, Boutoille D, Stindel E, Dinh A, Bemer P, Giraudeau B, Issartel B, Caille A (2021). Antibiotic Therapy for 6 or 12 Weeks for Prosthetic Joint Infection. N Engl J Med.

[bib1.bib7] Bessard G, Stahl JP, Dubois F, Gaillat J, Micoud M (1983). Modification de la pharmacocinetique de la doxycycline par l'administration de rif ampicine chez l'homme. Médecine Mal Infect.

[bib1.bib8] Bidell MR, Lodise TP (2021). Use of oral tetracyclines in the treatment of adult outpatients with skin and skin structure infections: Focus on doxycycline, minocycline, and omadacycline. Pharmacotherapy.

[bib1.bib9] Budge MD, Koch JA, Mandell JB, Cappellini AJ, Orr S, Patel S, Ma D, Nourie O, Brothers KM, Urish KL (2020). The In Vitro Efficacy of Doxycycline over Vancomycin and Penicillin in the Elimination of Cutibacterium Acnes Biofilm. Antimicrob Comb Devices.

[bib1.bib10] Bystedt H, Dornbusch K, Nord CE (1976). Concentrations of oxytetracycline, tetracycline and doxycycline in mandibular osteitis. Scand J Infect Dis.

[bib1.bib11] Ceccarelli G, Perciballi B, Russo A, Martini P, Marchetti F, Capparuccia MR, Iaiani G, Fabris S, Ciccozzi M, Villani C, Venditti M, D'Ettorre G, De Meo D (2023). Chronic Suppressive Antibiotic Treatment for Staphylococcal Bone and Joint Implant-Related Infections. Antibiot Basel Switz.

[bib1.bib12] Cerca N, Martins S, Cerca F, Jefferson KK, Pier GB, Oliveira R, Azeredo J (2005). Comparative assessment of antibiotic susceptibility of coagulase-negative staphylococci in biofilm versus planktonic culture as assessed by bacterial enumeration or rapid XTT colorimetry. J Antimicrob Chemoth.

[bib1.bib13] Citron DM, Tyrrell KL, Goldstein EJC (2014). Comparative in vitro activities of dalbavancin and seven comparator agents against 41 *Staphylococcus* species cultured from osteomyelitis infections and 18 VISA and hVISA strains. Diagn Micr Infec Dis.

[bib1.bib14] Clumeck N, Marcelis L, Amiri-Lamraski MH, Gordts B (1984). Treatment of severe staphylococcal infections with a rifampicin-minocycline association. J Antimicrob Chemoth.

[bib1.bib15] Colmenero JD, Fernández-Gallardo LC, Agúndez JA, Sedeño J, Benítez J, Valverde E (1994). Possible implications of doxycycline-rifampin interaction for treatment of brucellosis. Antimicrob Agents Chemother.

[bib1.bib16] Donahue HJ, Iijima K, Goligorsky MS, Rubin CT, Rifkin BR (1992). Regulation of cytoplasmic calcium concentration in tetracycline-treated osteoclasts. J Bone Miner Res.

[bib1.bib17] Dornbusch K (1976). The detection of doxycycline activity in human bone. Scand J Infect Dis.

[bib1.bib18] Doub JB, Nandi S, Putnam N (2022). Retention of Minocycline Susceptibility When Gram-Positive Periprosthetic Joint Infection Isolates Are Non-Susceptible to Doxycycline. Infect Dis Rep.

[bib1.bib19] Duployez C, Millière L, Senneville E, Piantoni L, Migaud H, Wallet F, Loïez C (2022). Evolution of antibiotic susceptibility profiles of staphylococci from osteoarticular infections: A 10-year retrospective study. Orthop Traumatol-Sur.

[bib1.bib20] El Helou OC, Berbari EF, Lahr BD, Eckel-Passow JE, Razonable RR, Sia IG, Virk A, Walker RC, Steckelberg JM, Wilson WR, Hanssen AD, Osmon DR (2010). Efficacy and safety of rifampin containing regimen for staphylococcal prosthetic joint infections treated with debridement and retention. Eur J Clin Micr Infec Dis Off Publ Eur Soc Clin Microbiol.

[bib1.bib21] Ertel-Pau V (2014). Prothèse de hanche ou de grnou : diagnostic et prise en charge de l’infection dans le mois suivant l’implantation, recommandations de la HAS.

[bib1.bib22] Fillingham YA, Della Valle CJ, Suleiman LI, Springer BD, Gehrke T, Bini SA, Segreti J, Chen AF, Goswami K, Tan TL, Shohat N, Diaz-Ledezma C, Schwartz AJ, Parvizi J (2019). Definition of Successful Infection Management and Guidelines for Reporting of Outcomes After Surgical Treatment of Periprosthetic Joint Infection: From the Workgroup of the Musculoskeletal Infection Society (MSIS). J Bone Joint Surg Am.

[bib1.bib23] Garraffo R, Lapalus P, Dellamonica P, Fournier JP, Bernard E (1988). The effect of rifampicin on the pharmacokinetics of doxycycline. Infection.

[bib1.bib24] Gnarpe H, Dornbusch K, Hägg O (1976). Doxycycline concentration levels in bone, soft tissue and serum after intravenous infusion of doxycycline. A clinical study. Scand J Infect Dis.

[bib1.bib25] Grossman TH (2016). Tetracycline Antibiotics and Resistance. Cold Spring Harb Perspect Med.

[bib1.bib26] Hamad T, Hellmark B, Nilsdotter-Augustinsson Å, Söderquist B (2015). Antibiotic susceptibility among Staphylococcus epidermidis isolated from prosthetic joint infections, with focus on doxycycline. APMIS.

[bib1.bib27] Jang TL, Hewlett A, Cortes-Penfield NW (2024). High Efficacy of Oral Tetracyclines in Prosthetic Joint Infection Treated With Debridement, Antibiotics, and Implant Retention (DAIR) or Resection Arthroplasty With Destination Spacer Placement. Cureus.

[bib1.bib28] Koch JA, Pust TM, Cappellini AJ, Mandell JB, Ma D, Shah NB, Brothers KM, Urish KL (2020). Staphylococcus epidermidis Biofilms Have a High Tolerance to Antibiotics in Periprosthetic Joint Infection. Life Basel Switz.

[bib1.bib29] Leijtens B, Weerwag L, Schreurs BW, Kullberg B-J, Rijnen W (2019). Clinical Outcome of Antibiotic Suppressive Therapy in Patients with a Prosthetic Joint Infection after Hip Replacement. J Bone Jt Infect.

[bib1.bib30] Lemaignen A, Bernard L, Marmor S, Ferry T, Grammatico-Guillon L, Astagneau P (2021). Epidemiology of complex bone and joint infections in France using a national registry: The CRIOAc network. J Infect.

[bib1.bib31] Li H-K, Rombach I, Zambellas R, Walker AS, McNally MA, Atkins BL, Lipsky BA, Hughes HC, Bose D, Kümin M, Scarborough C, Matthews PC, Brent AJ, Lomas J, Gundle R, Rogers M, Taylor A, Angus B, Byren I, Berendt AR, Warren A, Fitzgerald FE, Mack DJF, Hopkins S, Folb J, Reynolds HE, Moore E, Marshall J, Jenkins N, Moran CE, Woodhouse AF, Stafford S, Seaton RA, Vallance C, Hemsley CJ, Bisnauthsing K, Sandoe JAT, Aggarwal I, Ellis SC, Bunn DJ, Sutherland RK, Barlow G, Cooper C, Geue C, McMeekin N, Briggs AH, Sendi P, Khatamzas E, Wangrangsimakul T, Wong THN, Barrett LK, Alvand A, Old CF, Bostock J, Paul J, Cooke G, Thwaites GE, Bejon P, Scarborough M, OVIVA Trial Collaborators (2019). Oral versus Intravenous Antibiotics for Bone and Joint Infection. N Engl J Med.

[bib1.bib32] Mandell JB, Orr S, Koch J, Nourie B, Ma D, Bonar DD, Shah N, Urish KL (2019). Large variations in clinical antibiotic activity against Staphylococcus aureus biofilms of periprosthetic joint infection isolates. J Orthop Res.

[bib1.bib33] Matt M, Duran C, Courjon J, Lotte R, Moing VL, Monnin B, Pavese P, Chavanet P, Khatchatourian L, Tattevin P, Cattoir V, Lechiche C, Illes G, Lacassin-Beller F, Senneville E, Dinh A, Dalbavancin French Study Group (2021). Dalbavancin treatment for prosthetic joint infections in real-life: a national cohort study and literature review. J Glob Antimicrob Re.

[bib1.bib34] McNally M, Sousa R, Wouthuyzen-Bakker M, Chen AF, Soriano A, Vogely HC, Clauss M, Higuera CA, Trebše R (2021). The EBJIS definition of periprosthetic joint infection. Bone Joint J.

[bib1.bib35] Metsemakers WJ, Kuehl R, Moriarty TF, Richards RG, Verhofstad MHJ, Borens O, Kates S, Morgenstern M (2018). Infection after fracture fixation: Current surgical and microbiological concepts. Injury.

[bib1.bib36] Molina J-M, Charreau I, Chidiac C, Pialoux G, Cua E, Delaugerre C, Capitant C, Rojas-Castro D, Fonsart J, Bercot B, Bébéar C, Cotte L, Robineau O, Raffi F, Charbonneau P, Aslan A, Chas J, Niedbalski L, Spire B, Sagaon-Teyssier L, Carette D, Mestre SL, Doré V, Meyer L, Pintado C, Loze B, Gatey C, Ponscarme D, Penot P, Veron R, Delgado J, Dalle E, Parlier S, Madelaine I, Danet M, Mahjoub N, Mezreb N, Moudachirou K, Morel S, Conort G, Lorho F, Meunier M, Rozenbaum W, Monfort C, Foucoin J, Boissavy B, Cousseau S, Huon S, Danet M, Djessima A, Berrebi V, Adda A, Le Nagat S, Zarka L, Berdougo J, Mzoughi N, Clement F, Decouty A, Chapolard C, Godinot M, Adouard-groslafeige C, Koffi J, Pansu A, Becker A, Pailhes S, Bonnet F, Jeanblanc F, Brochier C, Teruin X, Rouby S, Gilly L, Etienne C, Tolonin F, Breaud S, Péchenot V, Bagge S, Cepitelli T, Roger Pm, Rosenthal E, Cheret A, Cornavin P, Vandamme S, Lambec J, Dumon N, Leclanche O, Huleux T, Biekre R, Melliez H, Bazus H, Pasquet A, Bernaud C, Besnier M, Bonnet B, Hall N, Cavellec M, Hue H, Larmet L, Colas M, Choquet R (2018). Post-exposure prophylaxis with doxycycline to prevent sexually transmitted infections in men who have sex with men: an open-label randomised substudy of the ANRS IPERGAY trial. Lancet Infect Dis.

[bib1.bib37] Monzón M, Oteiza C, Leiva J, Lamata M, Amorena B (2002). Biofilm testing of *Staphylococcus epidermidis* clinical isolates: low performance of vancomycin in relation to other antibiotics. Diagn Micr Infec Dis.

[bib1.bib38] Moore A, Ling M, Bucko A, Manna V, Rueda M-J (2015). Efficacy and Safety of Subantimicrobial Dose, Modified-Release Doxycycline 40 mg Versus Doxycycline 100 mg Versus Placebo for the treatment of Inflammatory Lesions in Moderate and Severe Acne: A Randomized, Double-Blinded, Controlled Study. J Drugs Dermatol.

[bib1.bib39] Nguyen S, Robineau O, Titecat M, Blondiaux N, Valette M, Loiez C, Beltrand E, Migaud H, Senneville E (2015). Influence of daily dosage and frequency of administration of rifampicin–levofloxacin therapy on tolerance and effectiveness in 154 patients treated for prosthetic joint infections. Eur J Clin Microbiol.

[bib1.bib40] Norden CW, Fierer J, Bryant RE (1983). Chronic staphylococcal osteomyelitis: treatment with regimens containing rifampin. Rev Infect Dis.

[bib1.bib41] Osmon DR, Berbari EF, Berendt AR, Lew D, Zimmerli W, Steckelberg JM, Rao N, Hanssen A, Wilson WR (2013). Diagnosis and Management of Prosthetic Joint Infection: Clinical Practice Guidelines by the Infectious Diseases Society of Americaa. Clin Infect Dis.

[bib1.bib42] Page MJ, McKenzie JE, Bossuyt PM, Boutron I, Hoffmann TC, Mulrow CD, Shamseer L, Tetzlaff JM, Akl EA, Brennan SE, Chou R, Glanville J, Grimshaw JM, Hróbjartsson A, Lalu MM, Li T, Loder EW, Mayo-Wilson E, McDonald S, McGuinness LA, Stewart LA, Thomas J, Tricco AC, Welch VA, Whiting P, Moher D (2021). The PRISMA 2020 statement: an updated guideline for reporting systematic reviews. BMJ.

[bib1.bib43] Parvizi J, Tan TL, Goswami K, Higuera C, Della Valle C, Chen AF, Shohat N (2018). The 2018 Definition of Periprosthetic Hip and Knee Infection: An Evidence-Based and Validated Criteria. J Arthroplasty.

[bib1.bib44] Perez-Alba E, Flores-Treviño S, Villarreal-Salazar V, Bocanegra-Ibarias P, Vilchez-Cavazos F, Camacho-Ortiz A (2023). Planktonic and biofilm states of *Staphylococcus aureus* isolated from bone and joint infections and the *in vitro* effect of orally available antibiotics. J Appl Microbiol.

[bib1.bib45] Pfaller MA, Flamm RK, Castanheira M, Sader HS, Mendes RE (2018). Dalbavancin in-vitro activity obtained against Gram-positive clinical isolates causing bone and joint infections in US and European hospitals (2011–2016). Int J Antimicrob Ag.

[bib1.bib46] Pimenta SP, Baldi BG, Kairalla RA, Carvalho CRR (2013). Doxiciclina em pacientes com linfangioleiomiomatose: biomarcadores e resposta funcional pulmonar. J Bras Pneumol.

[bib1.bib47] Pradier M, Nguyen S, Robineau O, Titecat M, Blondiaux N, Valette M, Loïez C, Beltrand E, Dézeque H, Migaud H, Senneville E (2017). Suppressive antibiotic therapy with oral doxycycline for Staphylococcus aureus prosthetic joint infection: a retrospective study of 39 patients. Int J Antimicrob Ag.

[bib1.bib48] Pradier M, Robineau O, Boucher A, Titecat M, Blondiaux N, Valette M, Loïez C, Beltrand E, Nguyen S, Dézeque H, Migaud H, Senneville E (2018). Suppressive antibiotic therapy with oral tetracyclines for prosthetic joint infections: a retrospective study of 78 patients. Infection.

[bib1.bib49] Preininger RE (1973). Treatment of chronic staphylococcal osteomyelitis with minocycline hydrochloride: a case report. Curr Ther Res Clin E.

[bib1.bib50] Prendki V, Zeller V, Passeron D, Desplaces N, Mamoudy P, Stirnemann J, Marmor S, Ziza J-M (2014). Outcome of patients over 80 years of age on prolonged suppressive antibiotic therapy for at least 6 months for prosthetic joint infection. Int J Infect Dis.

[bib1.bib51] Rao N, Crossett LS, Sinha RK, Le Frock JL (2003). Long-term suppression of infection in total joint arthroplasty. Clin Orthop.

[bib1.bib52] Ruhe JJ, Monson T, Bradsher RW, Menon A (2005). Use of long-acting tetracyclines for methicillin-resistant *Staphylococcus aureus* infections: case series and review of the literature. Clin Infect Dis Off Publ Infect Dis Soc Am.

[bib1.bib53] Sandiford NA, Hutt JR, Kendoff DO, Mitchell PA, Citak M, Granger L (2020). Prolonged suppressive antibiotic therapy is successful in the management of prosthetic joint infection. Eur J Orthop Surg Tr.

[bib1.bib54] Sato K, Yazawa H, Ikuma D, Maruyama T, Kajiyama H, Mimura T (2019). Osteomyelitis due to methicillin-resistant Staphylococcus aureus successfully treated by an oral combination of minocycline and trimethoprim-sulfamethoxazole. SAGE Open Med Case Rep.

[bib1.bib55] Segreti J, Nelson JA, Trenholme GM (1998). Prolonged suppressive antibiotic therapy for infected orthopedic prostheses. Clin Infect Dis Off Publ Infect Dis Soc Am.

[bib1.bib56] Shah NB, Hersh BL, Kreger A, Sayeed A, Bullock AG, Rothenberger SD, Klatt B, Hamlin B, Urish KL (2020). Benefits and Adverse Events Associated With Extended Antibiotic Use in Total Knee Arthroplasty Periprosthetic Joint Infection. Clin Infect Dis.

[bib1.bib57] Siqueira MBP, Saleh A, Klika AK, O'Rourke C, Schmitt S, Higuera CA, Barsoum WK (2015). Chronic Suppression of Periprosthetic Joint Infections with Oral Antibiotics Increases Infection-Free Survivorship. J Bone Joint Surg Am.

[bib1.bib58] Takahashi N, Ishihara K, Kimizuka R, Okuda K, Kato T (2006). The effects of tetracycline, minocycline, doxycycline and ofloxacin on *Prevotella intermedia* biofilm. Oral Microbiol Immun.

[bib1.bib59] Thiboutot DM, Fleischer AB, Del Rosso JQ, Rich P (2009). A multicenter study of topical azelaic acid 15 % gel in combination with oral doxycycline as initial therapy and azelaic acid 15 % gel as maintenance monotherapy. J Drugs Dermatol.

[bib1.bib60] Thompson JM, Saini V, Ashbaugh AG, Miller RJ, Ordonez AA, Ortines RV, Wang Y, Sterling RS, Jain SK, Miller LS (2017). Oral-Only Linezolid-Rifampin Is Highly Effective Compared with Other Antibiotics for Periprosthetic Joint Infection: Study of a Mouse Model. J Bone Joint Surg Am.

[bib1.bib61] Tonnelier M, Bouras A, Joseph C, Samad YE, Brunschweiler B, Schmit J-L, Mabille C, Lanoix J-P (2021). Impact of rifampicin dose in bone and joint prosthetic device infections due to *Staphylococcus spp*: a retrospective single-center study in France. BMC Infect Dis.

[bib1.bib62] Veerman K, Goosen J, Spijkers K, Jager N, Heesterbeek P, Telgt D (2023). Prolonged use of linezolid in bone and joint infections: a retrospective analysis of adverse effects. J Antimicrob Chemoth.

[bib1.bib63] Vollmer NJ, Rivera CG, Stevens RW, Oravec CP, Mara KC, Suh GA, Osmon DR, Beam EN, Abdel MP, Virk A (2021). Safety and Tolerability of Fluoroquinolones in Patients with Staphylococcal Periprosthetic Joint Infections. Clin Infect Dis.

[bib1.bib64] Warner AJ, Hathaway-Schrader JD, Lubker R, Davies C, Novince CM (2022). Tetracyclines and bone: Unclear actions with potentially lasting effects. Bone.

[bib1.bib65] Wouthuyzen-Bakker M, Nijman JM, Kampinga GA, van Assen S, Jutte PC (2017). Efficacy of Antibiotic Suppressive Therapy in Patients with a Prosthetic Joint Infection. J Bone Jt Infect.

